# Population structure within the one-dimensional range of a coastal plain katydid

**DOI:** 10.1371/journal.pone.0179361

**Published:** 2017-06-09

**Authors:** Gideon Ney, Johannes Schul

**Affiliations:** Division of Biological Sciences, University of Missouri, Columbia, Missouri, United States of America; National Cheng Kung University, TAIWAN

## Abstract

Biogeography plays a significant role in species’ dispersal, and in turn population structure, across the landscape. The North American katydid *Neoconocephalus melanorhinus* belongs to a genus with high mobility. Unlike other members of the genus, *N*. *melanorhinus* is a salt marsh specialist restricted to a narrow corridor along the Atlantic and Gulf coasts. In addition, their range crosses at least one known biogeographic barrier and possesses biogeographic characteristics of the stepping-stone as well as the hierarchical island model of dispersal. Using AFLP markers we searched for areas that conform to the predictions of isolation by distance and for areas of non-uniform increases in genetic variance, indicative of isolation by barrier. We found significant genetic differentiation between all twelve sampled sites. Isolation by distance was the predominant pattern of variation across their range. In addition, we saw possible evidence of two biogeographic barriers to gene flow, one at the Atlantic-Gulf divide and the other along the Gulf coast. We also observed a change in body size across the range. Body size, as measured by male hind femur length, correlated closely with latitude, a possible indication of differential selection across the species range.

## Introduction

The phylogeography of organisms, i.e. the distribution of genetic lineages of a species across landscapes [1], is strongly affected by their dispersal ability [2]. Isolation by distance (IBD) and isolation by barrier (IBB) are two common patterns found among numerous species and taxonomic groups [3]. Most species show dispersal limitations across some spatial scale [4], i.e. individuals cannot disperse across their entire species’ range during their lifetime. Such reduced dispersal can result in a pattern of uniformly increasing genetic variation with geographic distance (= IBD, [5]). Alternatively, IBB occurs if biogeographic barriers to dispersal reduce gene flow. This often leads to a pattern of genetic discontinuity, i.e. a non-uniform increase in genetic differentiation across the landscape [6]. While the difference between IBD and IBB is likely an artificial dichotomy, their study allows us to contemplate the relative importance that various factors play in shaping population structure.

A number of models explain the genetic structure resulting from different dispersal limitations [5, 6] such as distance or barriers. Within the stepping-stone model dispersal is limited by geographic distance and individuals disperse only between neighboring sites. In its most restricted form, sites fall along a line. Within this one-dimensional stepping-stone model, dispersal is limited to, at most, two adjacent sites. This produces a strong signature of IBD as gene flow decreases with distance along a narrow corridor [5, 7].

In an alternative model, the hierarchical island model, barriers between certain neighboring sites reduce the rate of dispersal and gene flow. This produces a non-uniform increase in genetic differentiation between these sites, the signature of IBB [6]. This divides the population structure into distinct subpopulations. Within subpopulations there is an assumption of little or no genetic differentiation (panmixia). This is the result of significant gene flow within, and reduced gene flow between, subpopulations. Unlike in the stepping-stone model, geographic distance does not play a role in this pattern of genetic differentiation.

*Neoconocephalus* is a diverse group of New World katydids with variation in habitat preference and species distributions [8]. All species are highly mobile with strong flying capabilities and adult life spans of several months (review in [8]). Most species occupy large ranges, some of which encompass much of the Midwestern and Eastern United States. The North American ranges of these species seem to show little population level genetic structure [9]. For example, *N*. *bivocatus* and *N*. *robustus* show a lack of genetic structure across more than 450 km of grassland habitat (G. Ney and J. Schul unpublished data).

**Fig 1:**
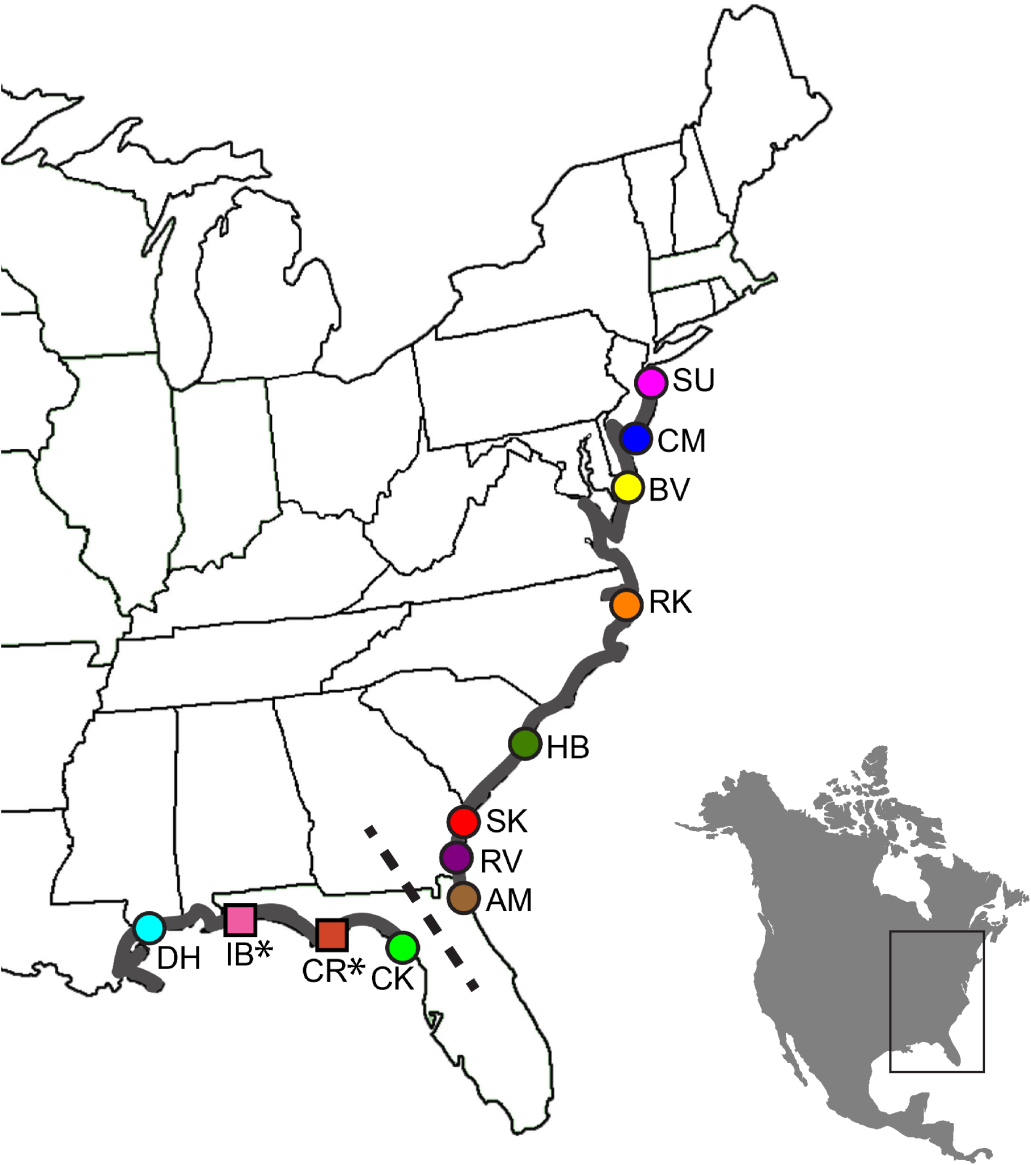
Map of collection localities. Shaded in area represents the hypothesized range of *N*. *melanorhinus* (modified from [12]) based on literature and collection records. Squares and * represent sites sampled only in 2015. The dashed line indicates the site of the hypothesized Atlantic-Gulf divide.

*Neoconocephalus melanorhinus* is a habitat specialist found only in North American salt marshes along the Atlantic and Gulf coasts [10]. This preference results in a species distribution, unique among *Neoconocephalus* katydids, that is more than 2,000 km long, but no more than a few kilometers wide in most localities. This one-dimensional arrangement of populations, which is similar to those in a one-dimensional stepping-stone model, may limit dispersal to neighboring sites and reduce gene flow across the range. *Neoconocephalus melanorhinus’* range also falls along several known biogeographic divides, including one dividing the range on either side of the Florida peninsula (Fig 1). This Atlantic-Gulf divide is a significant barrier to gene flow in many marine taxa found along the Southern United States coast [3, 11]. In addition, their range crosses numerous other geographic features, including estuaries, developed shoreline, and stretches of coastline lacking saltmarsh habitat, that could potentially function as biogeographic barriers. *Neoconocephalus melanorhinus’* narrow range may decrease individuals’ ability to avoid barriers by going around them and result in greater genetic differentiation among subpopulations on either side.

Here we describe the population genetic structure of *N*. *melanorhinus*, a highly mobile species, in a one-dimensional range, which possesses characteristics of both a stepping-stone and a hierarchical island model. We sampled from twelve locales across the range. Using molecular markers and statistical tools, we searched for areas that conform to the predictions of IBD and for areas with non-uniform increases in genetic variance indicative of IBB. We note the confounding effect signals of IBD and IBB may have and utilized tools that control for these effects. In addition, we collected measurements for body size to determine whether this phenotype co-varies with the observed pattern of genetic differentiation across *N*. *melanorhinus’* range.

## Materials and methods

### Sampling

All necessary permissions were obtained prior to the collection of specimens (Cape May NWR; permit 14007). We collected a total of 227 *N*. *melanorhinus* from 12 salt marshes sites (Table 1, Fig 1) along the US Atlantic and Gulf coasts. We localized males using their calls and collected them by hand within the 2–3 hours following sunset. Calling activity was greatest at dusk, with individuals ceasing calling activity within an hour of sunset. *Neoconocephalus melanorhinus* were found either on or in close proximity to *Juncus roemerianus* in the high marsh habitats South of the Chesapeake Bay. In the three most Northern sites (BV, CM, and SU) *J*. *roemerianus* was less common and individuals were predominantly found calling from cord grass. We identified individuals as belonging to the target species through their call and morphological features, including cone pigmentation and body shape [10, 12].

**Table 1:** *Neoconocephalus melanorhinus* sample list.

Sampling site	Code	Latitude	Longitude	Shoreline	N	PL 5%	Hj
**2014**							
Diamond Head, MS	DH	30.363	-89.375	0.0	8	1205	0.193
Cedar Key, FL	CK	29.210	-83.064	622.3	18	1119	0.184
Amelia Island, FL	AM	30.630	-81.473	842.3	25	1025	0.172
Ridgeville, GA	RV	31.407	-81.396	929.1	23	1027	0.178
Skidaway Island, GA	SK	31.948	-81.055	997.3	21	1140	0.176
Huntington Beach, SC	HB	33.513	-79.057	1252.6	19	1091	0.170
Roanoke Island, NC	RK	35.838	-75.620	1659.6	27	1023	0.166
Bishopville, MD	BV	38.404	-75.125	1948.2	9	1125	0.174
Cape May Court House, NJ	CM	39.108	-74.891	2029.1	22	1103	0.168
Surf City, NJ	SU	39.685	-74.208	2116.0	16	1061	0.174
**2015**							
Diamond Head, MS	DH	** Eight samples collected in 2014			9	728	0.218
Indian Bayou, Milton, FL	IB	30.528	-87.101	218.8	13	689	0.237
Carrabelle, FL	CR	29.845	-84.670	456.3	25	705	0.224
Cedar Key, FL	CK	** All samples collected in 2014			10	731	0.203

Table includes samples from 2014 and 2015, sampling site, locality code, latitude, longitude, relative simplified shoreline distance from site DH (km), sample size (N), polymorphic loci at 5% level (PL 5%), and expected heterozygosity under Hardy-Weinberg genotype proportions (Hj). Simplified shoreline distance is measured as the distance along a path connecting neighboring sites by straight lines.

(**) Eight DH samples and all ten CK samples from 2014 were added to the 2015 dataset (reflected in sample sizes listed).

In 2014, we collected 188 *N*. *melanorhinus* from all the Atlantic coast sites and the Gulf coast sites DH and CK (Table 1, Fig 1). After finding evidence of significant genetic differentiation along the Gulf, two additional sites were sampled (IB and CR) in 2015. One additional sample was added to site DH in 2015 as well. The thirty-nine samples added in 2015 were run with a subset of the 2014 samples (eight DH, ten CK), and of these, eight 2014 samples were run in replicate in 2015 (see below).

We removed one hind femur from each *N*. *melanorhinus* sampled and placed them in 95% EtOH for DNA preservation. We later extracted DNA from the hind femurs using the DNeasy Blood and Tissue Kit (Qiagen Inc., Valencia, CA, USA). DNA quantification was performed on each sample by spectrophotometry (NanoDrop 1000, Thermo Scientific, Wilmington, DE). We used amplified fragment length polymorphism (AFLP) markers to quantify genetic variation.

### Quantification of AFLP loci and alleles

AFLP bands were generated using the method described in Snyder et al. [13]. Briefly, we performed a double digest on whole genomic material using the enzymes EcoRI and MseI, followed by ligation of adapters onto the fragments’ sticky ends. We then performed two rounds of selective amplification. Pre-selective polymerase chain reaction (PCR) was carried out using primers Eco+A and Mse+A [13]. We performed selective PCR with one of four MseI primers (Mse+ATA, Mse+AGA, Mse+AAC, Mse+AGC) and one of two fluorescently labeled EcoRI primers (Eco+AAC, 6FAM; Eco+AGC, PET) (as described in [13]). The selective PCR products were genotyped at the University of Missouri’s DNA Core Facility on an ABI 3730 genetic analyzer (Applied Biosystems Corporation, Foster City, CA, USA). We called AFLP bands using GeneMarker v1.6 (Softgenetic Corp, State College, PA, USA) using an automated peak-calling scheme (as described in [14]). Alleles were called between 50–500 bp with a minimum peak intensity of 50.

### AFLP error analysis

We tested the rate of genotyping errors between collection years to determine the viability of combining AFLP datasets. In order to test repeatability, eight 2014 samples were rerun in replicate with the 2015 samples. Fragment lengths were recalled for the original as well as both sets of rerun samples as described previously and the mismatch error rate was calculated within and between AFLP runs [15]. Mismatch error rates between-runs in excess of within-run error rates would indicate additional between-run biases in fragment amplification or fragment calling, suggesting the need for an independent treatment of datasets [16].

The 2014 AFLP analysis produced 2170 distinct fragments from 188 individuals. The 2015 AFLP run produced 1175 fragments from fifty-seven individuals. Mismatch error rates averaged 6.1% for within-run peak calls and 14.0% for between-run peak calls. Because of the increase in mismatch error rates between AFLP runs we chose to not combine 2014 and 2015 datasets. By treating datasets independently we avoid added error incorporated by uncontrolled variations in fragment amplification or peak intensities between-runs.

Automated peak calling schemes produce a larger number of total fragments, but tend to produce larger error rates than manual peak calling schemes. Larger AFLP datasets, despite added noise, reveal greater genetic differentiation than smaller, less error prone, datasets [17]. As genetic variation tends to be relatively low within *Neoconocephalus*, the automated peak-calling scheme is preferable due to its sensitivity in detecting lower levels of genetic variation. Significant patterns of genetic structure found in this study would indicate that these levels of within-run noise do not obscure the overall genetic signal.

### Phylogenetic analysis

A fifty percent rule Bayesian tree was constructed in MrBayes v.3.2 from individuals collected in 2014 [18]. The MCMC settings for the MrBayes analysis were: two runs, each with eight chains for 20,000,000 generations, sampled every 10,000 generations, and sampled for a total of 2,000 trees per run. As AFLP fragments are dominant markers scored as present/absent, we utilized the binary restriction site model and the ‘noabsencesites’ substitution model. Runs were determined to have reached a stationary distribution by viewing the log-likelihoods in Tracer v.1.5 [19].

### Genetic differentiation

We used four methods to quantify genetic differentiation across *N*. *melanorhinus’* range. First, genetic differentiation among localities was calculated using a Bayesian non-uniform allele frequency distribution method for each year’s dataset independently [20] as implemented in the program AFLPsurv v1.0 [21]. Expected F_ST_ was estimated from 100,000 permutations (10 bootstrapped runs) of individuals among groups.

Second, a Bayesian clustering analysis of individuals from 2014, as implemented in the program STRUCTURE v.2.3.4 [22], produced assignment scores for individuals to a hypothesized number of clusters based on AFLP fragment data. In STRUCTURE the admixture model was implemented with a local prior, allele frequencies correlated, with a run length of 100,000 (Burnin = 10,000) for 10 replicates each of K = 1–12. The best supported K was estimated as described in [23] and implemented in Structure Harvester v.0.6.94 [24]. The program Clumpp v.1.1.2 [25] was used to align the 10 repetitions of the best supported number of clusters.

Third, multiple AMOVAs were performed using the 2014 and 2015 AFLP datasets. AMOVA is a method for testing hypotheses of population structure against genetic evidence [26, 27]. Sites were split into two groups at every possible location along the coast resulting in nine AMOVAs among the ten sites sampled in 2014 and three AMOVAs among the four Gulf coast sites from the 2015 dataset. AMOVAs were conducted in Arlequin v.3.5 [28] using the locus-by-locus approach, averaged over all polymorphic loci.

Fourth, standard and partial Mantel tests were conducted for populations sampled in 2014 using the program GenoDive v.2.0 [29] to examine the relationship between genetic differentiation (F_ST_/(1-F_ST_)), genetic structure, and geographic distance. Geographic distance was measured as the simplified shoreline distance, the distance along a path connecting neighboring sites by straight lines. This path is likely similar to the path *N*. *melanorhinus* must disperse through, not necessarily following all of the contours of the coast, but also not crossing large stretches of non-marsh habitat. In addition, we conducted a linear model analysis of variance in R v.3.2.0 [30] to test for differences in dispersal across predicted barrier sites by testing for significant differences in slopes and y-intercepts.

We produced three matrices representing the most well supported models of population structure formulated from the other analyses. Significance was calculated by 1,000 permutations of samples between groups. To directly take into account population structure, partial Mantel tests were performed measuring the relationship between genetic and geographic distance, while using the hypothesized genetic clustering as a covariate. In addition, partial Mantel tests examining the relationship between genetic differentiation and the three hypothesized population structures were run with geographic (simplified shoreline) distance as a covariate.

### Phenotypic differentiation

We measured body size using the length of the hind femur. After removing a single hind leg from each sampled individual, the length from the proximal end of the leg to the first joint was measured using a ruler to the nearest half-millimeter. As there is sexual dimorphism in *Neoconocephalus*, only the body measures from males were used in this analysis. In addition, as there may be an effect between years, we chose to compare only males collected in 2014. Hind femur length was correlated with latitude and the significance evaluated using Pearson’s correlation coefficient. In addition, the difference in average male hind femur length between sites was correlated with genetic differentiation and evaluated using a Mantel test.

## Results

### Phylogenetic analysis

**Fig 2:**
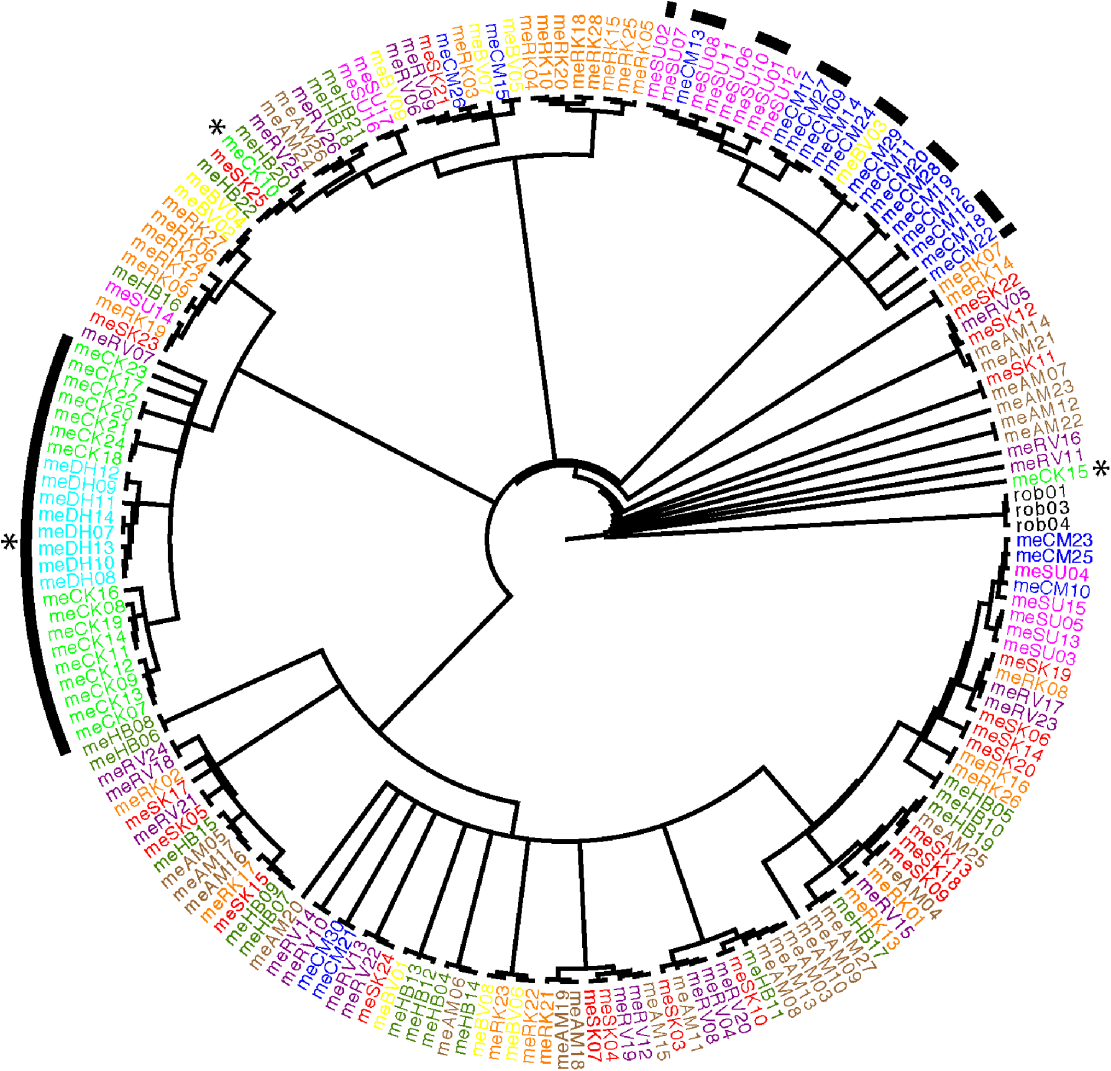
Fifty percent rule Bayesian tree of 2014 individuals. Tree was constructed using MrBayes v.3.2 (2 runs, 3 chains, and 20 million generations). Three samples of *N*. *robustus* were used as an out-group. Site localities are indicated by colors corresponding to Fig 1 and site abbreviation in taxa labels. * Indicates Gulf coast individuals; solid line indicates a clade consisting entirely of Gulf coast individuals; dashed line indicates the large North Atlantic clade.

A phylogenetic analysis of individuals collected from all sites in 2014 allowed us to determine a hypothesis of the ancestry among all individuals. Sites whose individuals share ancestry with individuals from other sites have likely seen significant gene flow in their past. Sites comprised of monophyletic clusters of individuals have likely remained isolated and have an ancestry less affected by gene flow. Individuals from South Atlantic sites did not form a monophyletic clade, but a large clade made up almost exclusively of North Atlantic (sites SU and CM) individuals was nested within the larger cluster of South Atlantic sites (Fig 2). This suggests that range expansion likely proceeded South to North along the Atlantic coast. The presence of individuals from the two Northern sites (SU and CM) intermixed with individuals from more Southern sites may indicate the presence of a genetic source population in the North Atlantic, from which individuals may emigrate, but to which Southern individuals do not commonly immigrate.

All but two individuals from the Gulf coast sites (DH, CK) formed a monophyletic clade (Fig 2), nested within the larger Atlantic cluster of animals. This suggests that the Gulf population was likely established following a dispersal event from an ancestral Atlantic population. The Atlantic-Gulf divide likely acts as a barrier, reducing gene flow and maintaining genetic differentiation following dispersal to the Gulf [11]. Two CK individuals, however, fell outside of the Gulf clade and may represent recent migrants from the Atlantic. This suggests limited recent dispersal across the Atlantic-Gulf divide. Within the Gulf clade, individuals from the Western Gulf site DH all fell into a monophyletic cluster. The monophyletic clustering of individuals by site within both Gulf sites suggests the presence of reduced gene flow via IBB. A pattern of IBD better explains the intermixing of individuals observed between neighboring sites along the South Atlantic portion of the range.

### Bayesian clustering analysis

**Fig 3:**
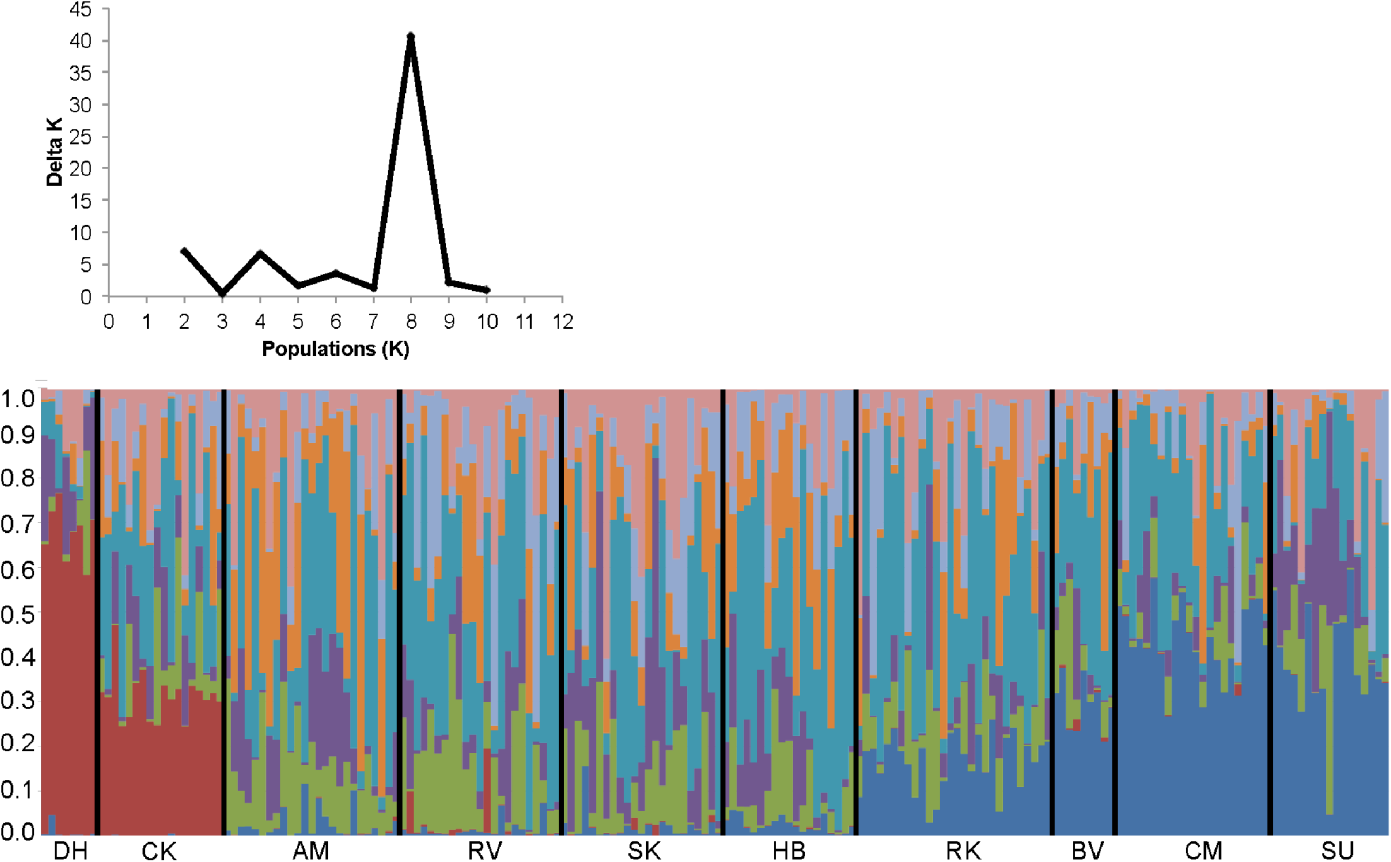
Consensus shared ancestry population structure for the 2014 dataset (K = 8). (Top) Delta K calculated using the Evanno et al. (2005) method. The modal value of this distribution is the accepted K or the uppermost level of structure, here K = 8 genetic clusters. (Bottom) Each bar represents an individual grouped by sampling sites. Sites are listed next to their nearest neighbors (West to East; South to North).

Our Bayesian clustering analysis, utilized to independently evaluate the grouping of individuals seen in the phylogeny, revealed a peak ΔK [23] for K = 8 genetic clusters among the 2014 individuals (Fig 3). We observed one genotype cluster found almost exclusively among individuals from the two Gulf sites (red in Fig 3). The lack of this genetic cluster among Atlantic individuals is an indication of hierarchical genetic differentiation between Gulf and Atlantic sites. Reduced dispersal across the Atlantic-Gulf divide is the most likely cause for this pattern.

We observed a second cluster (dark blue in Fig 3) mainly to individuals from Northern Atlantic sites. Its occurrence decreases to the South. This pattern may indicate the presence of a genetic cline and continued gene flow, rather than a barrier within the Atlantic range. The other six clusters did not assign to individuals from any particular site or region (Fig 3). The results of the STRUCTURE analysis showed support for the significance of the Atlantic-Gulf divide on *N*. *melanorhinus’* population structure, as well as an additional barrier along the Gulf. Genetic differentiation along the Atlantic appears to be clinal, with IBD alone shaping genetic structure.

### AMOVA test of population structure

**Fig 4:**
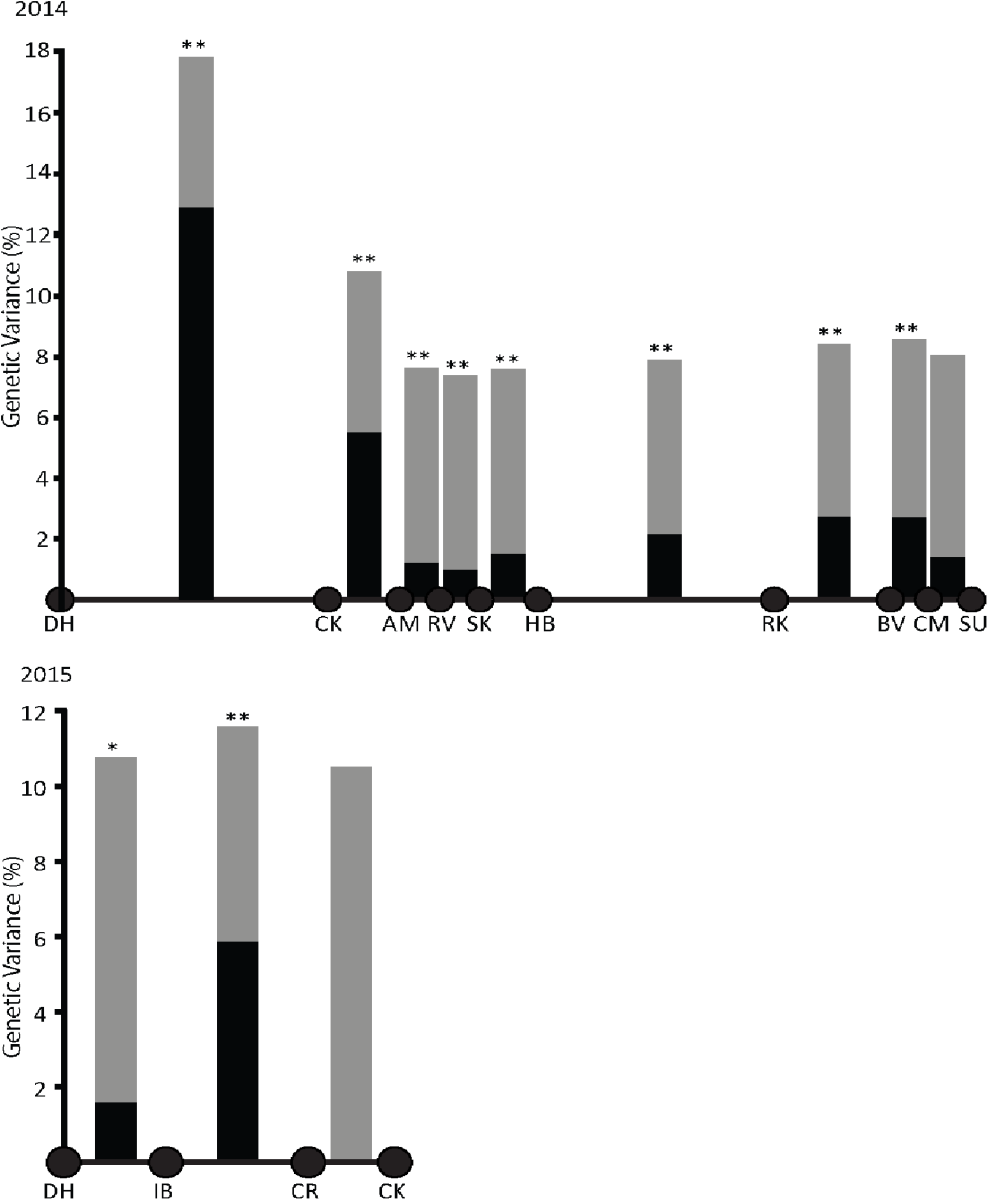
Locus-by-locus analysis of molecular variance (AMOVA) conducted grouping sites into adjacent groups at each site along the coast. AMOVAs are shown for 2014 and 2015 datasets. Bars indicate the results of an AMOVA conducted by grouping sites on either side of the bar into two separate groups. Black bars represent the genetic variance explained among hypothesized groups split between those two localities and gray bars the genetic variance explained within these groups. Significance of among-group genetic variance sums of squares indicated as * (p < 0.05) or ** (p < 0.00001). Among-group genetic variance was negative for CR to CK in 2015, shown as zero.

To test our hypotheses of population structure against genetic differentiation, we tested for the grouping of sites into subpopulations, as would be expected under a model of IBB. Twelve hypothetical genetic structures were produced by dividing sites into two subpopulations at each break between sampled sites. Each hypothetical population structure, implemented from 2014 and 2015 samples, was tested using a separate AMOVA. For the 2014 dataset, among-group genetic variance was highest when Gulf site DH was assigned to one group and all other locations to another (12.86%, Fig 4). Thus, DH was genetically distinct from all the other sites and dispersal was limited along the Gulf coast. The addition of two Gulf sites in 2015 allowed us to determine that most of the Gulf’s genetic variance is the result of reduced dispersal between sites IB and CR (5.88% among group variance, Fig 4). This pointed to the presence of a barrier between these sites, rather than a gradual increase in genetic variance with distance across the length of the Gulf coast, as would be expected under IBD.

In addition, we observed a relatively high level of among-group genetic variance at the site of the Atlantic-Gulf divide. All genetic structures subdividing sites along the Atlantic explained relatively little among-group variance. These patterns of genetic variance indicated that biogeographic barriers occurred between Gulf sites IB and CR, and at the Atlantic-Gulf divide.

### Mantel tests of population genetic versus geographic distances

**Fig 5:**
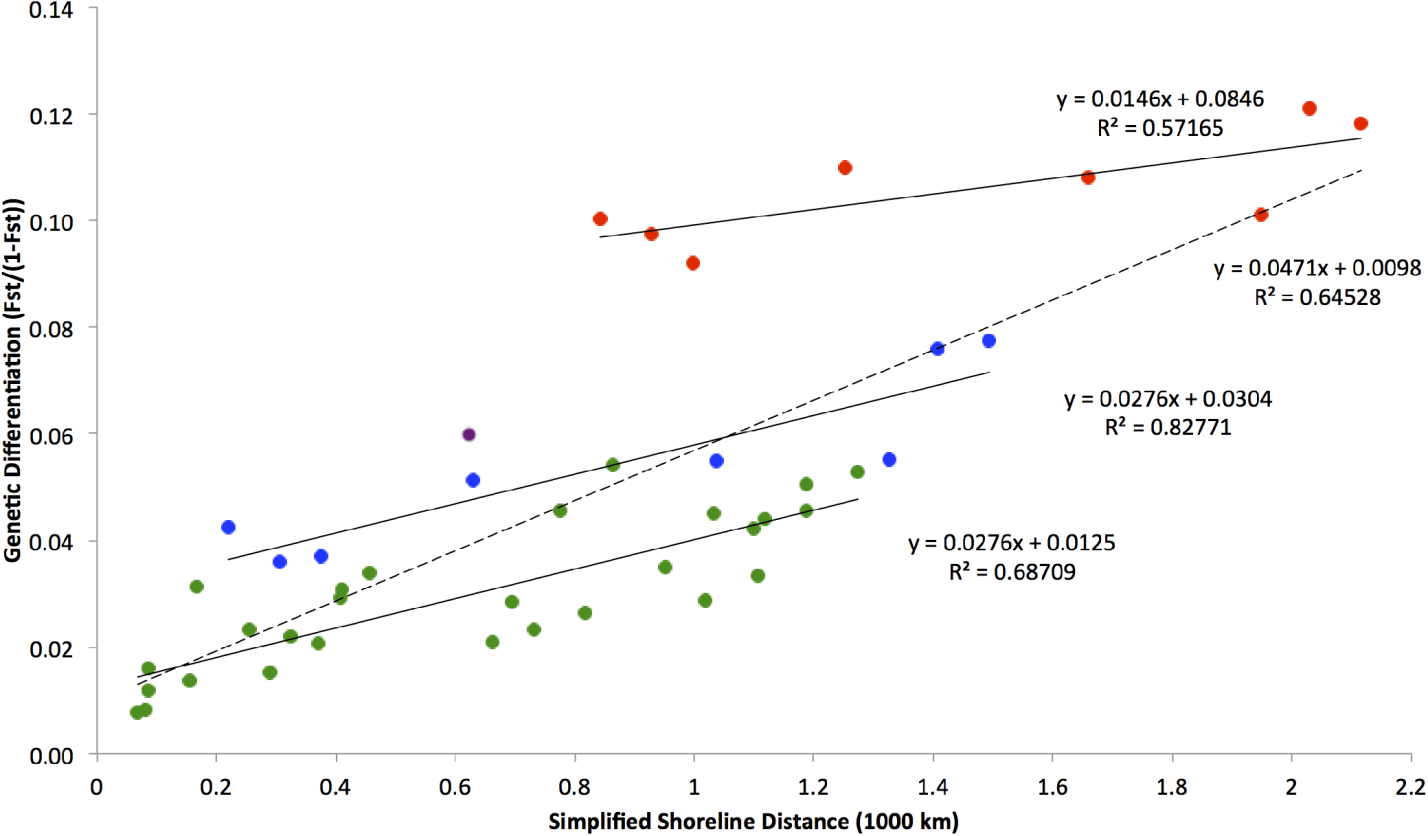
Relationship between genetic differentiation and geographic distance within 2014 dataset. Between-Atlantic site comparisons (green), between CK and Atlantic sites (blue), and between DH and Atlantic sites (red). Purple indicates the pairwise differences between Gulf sites DH and CK (the westernmost and easternmost Gulf coast sites). The dotted line represents the linear correlation between all points. The equation and R^2^ are provided for each line.

To examine the relationship between population genetic distance and geographic distance, we examined the correlation between genetic differentiation (F_ST_/(1- F_ST_)) and geographic distance, often used as an indication of IBD, and found a significant correlation (Mantel R = 0.795, p < 0.001) among the 2014 sites (Table 2). We subdivided pairwise F_ST_ values (S1 Table) into three groups based on sampling localities: within-Atlantic pairs (no barriers between, as indicated by previous analyses), Atlantic v. CK pairs (one barrier between), and Atlantic v. DH pairs (two barriers between). Within all three groups, genetic variation increased with geographic distance (Fig 5).

**Table 2:** Results of standard and partial Mantel tests distinguishing the effects of the 2014 dataset’s genetic differentiation, geographic distance, and population clustering.

Matrix A	Matrix B	Adjustment	Mantel’s R	P value
1) Genetic	Geographic distance	-	0.795	0.001
2) Genetic	Gulf and Atlantic-Gulf divide clusters	-	0.891	0.009
3) Genetic	Gulf divide only clusters	-	0.832	0.074
4) Genetic	Atlantic-Gulf divide only clusters	-	0.750	0.027
5) Genetic	Geographic distance	Partial: Gulf and Atlantic-Gulf divide clusters as covariate	0.812	0.001
6) Genetic	Gulf and Atlantic-Gulf divide clusters	Partial: geographic distance as covariate	0.894	0.748
7) Genetic	Geographic distance	Partial: Gulf divide only clusters as covariate	0.784	0.003
8) Genetic	Gulf divide only clusters	Partial: geographic distance as covariate	0.813	0.751
9) Genetic	Geographic distance	Partial: Atlantic-Gulf divide only clusters as covariate	0.773	0.001
10) Genetic	Atlantic-Gulf divide only clusters	Partial: geographic distance as covariate	0.705	0.753

P values are estimated from 1,000 permutations of samples within matrices.

The slopes of the three linear correlations did not differ significantly from one another, based on a linear model analysis of variance. However, based on linear models, the groups’ linear trend lines differed significantly in their y-intercepts, with the within-Atlantic line having the smallest and DH v. Atlantic the largest y-intercept. This evidence suggests the existence of barriers along the coast at the sites of both the proposed Atlantic-Gulf and Gulf divides. The similarity in slopes among the three subgroups indicates an impact of IBD across the entire range. We also examined the relationship between genetic differentiation and latitude. While this relationship was significant (Mantel R = 0.3793; p = 0.010), geographic distance described more of the variation in genetic differentiation between sites.

We utilized Mantel tests to determine the significance of correlations between genetic distance, geographic distance, and several hypothesized population structures. We produced three hypothesized population structures based on the presence of one or both hypothesized barriers (Atlantic-Gulf divide and/or within Gulf barrier). We observed a significant correlation between genetic differentiation and the population structure model built with both barriers (Table 2 row 2). The Atlantic-Gulf divide only hypothesis also showed a significant correlation with genetic differentiation (Table 2 row 4), however the Gulf divide only hypothesis did not meet significance with a p < 0.05 criterion (Table 2 row 3).

Because of these significant correlations of genetic differentiation with both distance and hierarchical population structures described above, we performed several partial Mantel tests to control for the confounding effects between them. Partial Mantels allowed for the comparison of two variables, while controlling for the effects of a third. Genetic differentiation and geographic distance maintained a significant relationship when population structure was controlled for (Table 2 rows 5,7,9). However, the correlation between genetic differentiation and the hypothesized population structures lost significance when geographic distance was controlled for (Table 2 rows 6,8,10). The partial Mantel analyses indicated that a prediction of IBD alone was better supported than any of our hierarchical population structures.

### Phenotypic differentiation

**Fig 6:**
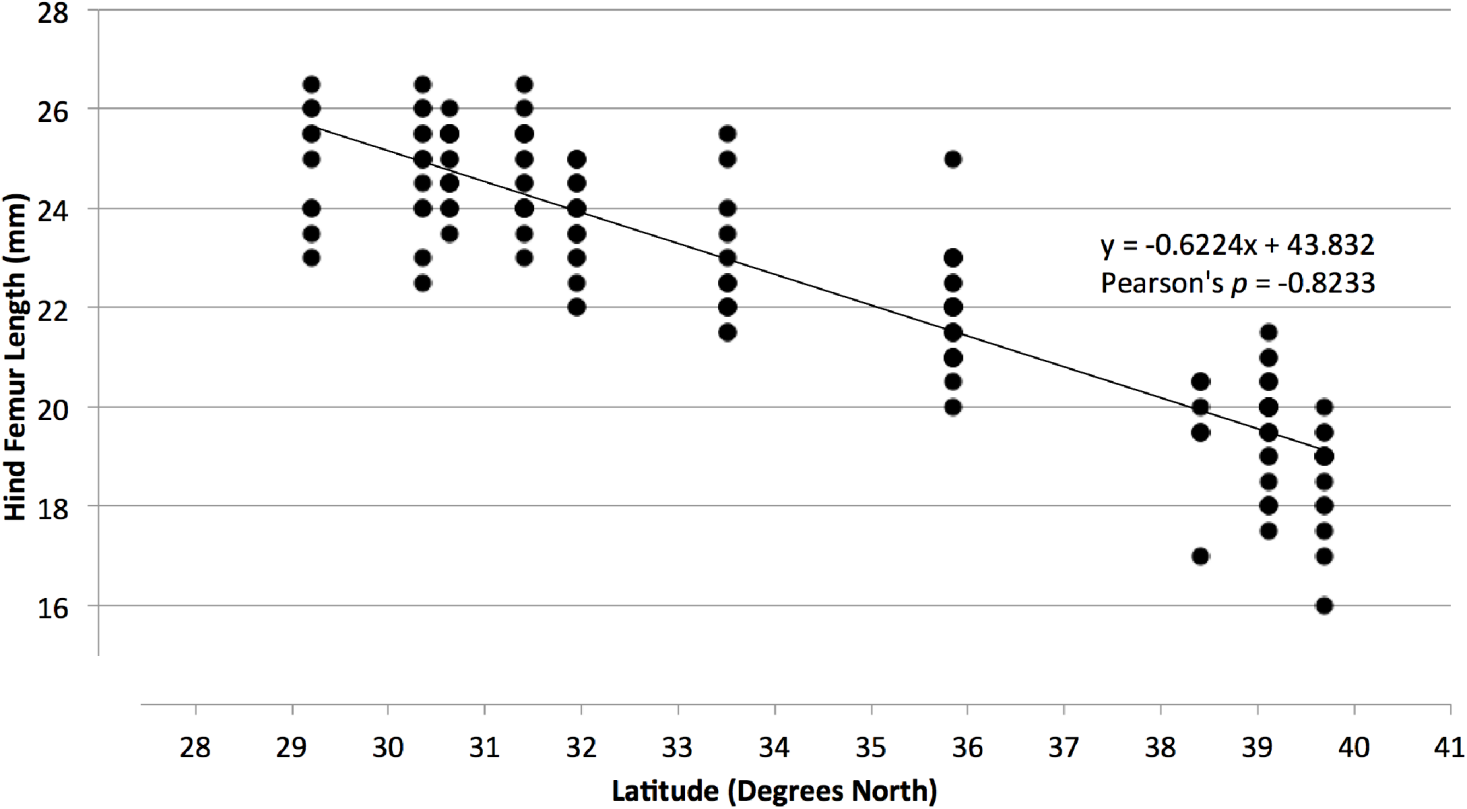
Relationship between hind femur length and latitude among 2014 males. The equation of the line of best fit and the Pearson’s correlation coefficient are provided.

We observed a change in body size across *N*. *melanorhinus’* range. Hind femur length showed a strong linear relationship with latitude (Fig 6, Pearson’s p = -0.8233). Gulf and South Atlantic individuals, found at similar latitudes, were of a similar size. On the other hand, phenotypic variation, as measured by the difference in average male hind femur length, was not significantly correlated with genetic differentiation (Mantel R = 0.297, p = 0.074).

## Discussion

*Neoconocephalus melanorhinus* showed significant genetic differentiation among all sampled sites. IBD was the predominant pattern of variation across the range. However, some of our analyses found evidence for at least two biogeographic barriers to gene flow; at the site of the Atlantic-Gulf divide and a second along the Gulf coast, resulting in a hierarchical genetic structure.

### Genetic isolation by distance

We observed a relationship between genetic differentiation (F_ST_/(1-F_ST_)) and geographic distance along both the Atlantic and Gulf coasts (Fig 5). All Mantel tests comparing genetic to geographic distances showed significance, an indication of IBD (Fig 5, Table 2). In most cases neighboring sites were the most closely related, resulting in a genetic cline along the coast. This would indicate that *N*. *melanorhinus* follows a stepping-stone like model of dispersal [5, 7]. *Neoconocephalus melanorhinus’* largely one-dimensional distribution limits movement to corridors connecting neighboring sites, decreasing dispersal to the least number of paths possible, and reducing gene flow at greater distances.

Ecological differences may also increase with increasing geographic distance, especially with changing latitude along the Atlantic coast. The observed genetic differentiation could be due to differences in selection, resulting in partial reproductive isolation between locally adapted populations. Determining whether reproductive isolation correlates more closely with geographic distance or microhabitat could differentiate these alternative explanations. This, however, could be difficult as distance and ecological differences are themselves correlated [31].

In the case of *N*. *melanorhinus*, genetic differentiation increases both along the North/South axis of the range, where variation in ecology is likely to be greatest, and also along their East/West axis (at its southernmost limit), where many ecological factors, especially those correlated with season length or temperature are likely to be more conserved. As such, geographic distance explained genetic differentiation (Fig 5) better than latitude alone. Further analyses investigating specific ecological differences and their correlation with genetic differentiation may be a worthwhile area of future investigation.

### Hierarchical genetic structure

We found evidence for two barriers to gene flow in the phylogenetic pattern (Fig 2), the STRUCTURE analysis (Fig 3), and the Mantel test (Fig 4), while the partial Mantel tests (Table 2) failed to detect these barriers. Below we argue that this failure of the partial Mantel tests is an artifact of our sampling scheme (see section *Correcting for relative effects of IBD and IBB)*.

Increased genetic differentiation in vertebrates, invertebrates, and plant species occurs along the Atlantic and Gulf coasts, specifically at the site of the Atlantic-Gulf divide (reviewed in [3, 11]). The fact that so many diverse taxa (e.g. horseshoe crab [32], dusky seaside sparrow [33], and diamond-back terrapin [34]) show similar phylogeographic patterns across this region likely indicates a shared history. While no single geologic or environmental event has been identified, changing environmental conditions and the shifting presence of tropical and subtropical habitats in this region during the Pleistocene likely have played a substantial role in population level diversification in the region [3].

The contemporary lack of obligatory salt marsh habitat along most of the Florida peninsula seems a plausible barrier to dispersal for *N*. *melanorhinus*. Contemporary salt marsh habitats became established in North America in the past 3,000 to 4,000 years [35]. Given *N*. *melanorhinus’* single generation per year, the recent biogeography of the region is the most likely source of their population structure. Thus, we assume that contemporary biogeographic factors were sufficient to produce the patterns of IBB observed in *N*. *melanorhinus*.

In addition to the Atlantic-Gulf divide, we found evidence of a second barrier to dispersal along the Florida panhandle between localities IB and CR. This stretch of coast possesses few barrier islands and reduced salt marsh habitat. The salt marsh habitat that is present is primarily fragmented patches found upstream in estuaries. It is likely that the reduced connectivity of salt marsh habitat along the Gulf has resulted in the observed pattern of genetic differentiation.

### Correcting for relative effects of IBD and IBB

The ability to differentiate the affects of IBD and IBB is important for the proper interpretation of population structure. Some of the most commonly used tools (e.g. STRUCTURE) to investigate patterns of hierarchical genetic clustering may falsely detect hierarchical population structure in datasets influenced only by IBD [36, 37, 38, 39]. Similarly, datasets with only hierarchical genetic structure can show results indicative of IBD (standard Mantel tests, Fig 5) [39].

Partial Mantel tests were used to separate the affects of IBD and IBB on *N*. *melanohrinus’* observed genetic variance. The results of the partial Mantel tests indicated that a pattern of IBD alone explained a significant proportion of the genetic variation. However the partial Mantel tests did not indicate an affect of IBB. A result that contradicted the findings of several of our previous analyses (e.g. phylogeny, AMOVA, structure, and stratified Mantel) that indicated hierarchical genetic structure explained a large proportion of the total genetic variance. We find it likely that the partial Mantel test failed to find the true signal of IBB where the other analyses did, as apposed to a type one error being present in all the other analyses. Multiple facets of this study’s sampling scheme may have made the detection of a signal of IBB difficult.

Inconsistences between our sampled data and our model of IBB may have led to type two errors in the partial Mantel tests. Within-group variance, like that observed across the Atlantic range, has a significant impact on *N*. *melanorhinus’* genetic structure and may have obscured a signal of between-group variance [36, 40]. The partial Mantel test may have therefore failed to find support for IBB because excessive within-group variance broke the assumption of within-subpopulation panmixia, suggestive of a pattern of hierarchical genetic structure [40]. In addition, the uneven sampling of sites from the Atlantic and Gulf coasts, likely exaggerated the skewed variance between subpopulations. Because of the likely bias in genetic variance between subpopulations and its affect on the partial Mantel, we conclude that both IBB and IBD still likely play a role in the observed pattern of genetic structure.

*Neoconocephalus melanorhinus’* strong signal of IBD is likely, at least in part, the result of its strict habitat specificity and the linear arrangement of its coastal habitat. Habitat specialists often show decreased dispersal across complex, heterogeneous landscapes because of the uneven distribution of their resources [41]. Populations surrounded by largely inhospitable environments are likely to incur higher “risk costs” [42], as the likelihood of locating required resources at neighboring sites is low penalizing dispersal. Higher risk costs are likely to increase the selective pressure against dispersal, even in highly mobile species with the physiological capability for long distance dispersal [43].

### Phenotypic variation

We observed significant correlation between hind femur length and latitude. This variation in body size could be due to differences in either genetic or environmental effects. Ectotherms, reliant on a limited warm period for development, grow larger at lower latitudes where the summer seasons are longer ("the converse of Bergmann’s rule" [44, 45]). When raised in common garden conditions, populations of Orthopterans tend to show the same pattern of differential growth as wild caught populations [46, 47, 48, 49, 50]. This indicates a genetic mechanism underlying this pattern [45]. However, we observed no significant relationship between body size and overall genetic differentiation between collection sites. The AFLP dataset used in this study represents a genome-wide measure of genetic variation. Specific genes responsible for size determination could show a divergent pattern of variance from the genome as a whole if under selection. A similar common garden experiment with *N*. *melanorhinus* could clarify the genetic contribution to size determination.

### Conclusions

*Neoconocephalus melanorhinus*’ population structure showed characteristics in common with both the stepping-stone and hierarchical island (barrier) models. While IBD has a larger overall effect, biogeographic barriers have a measureable impact on genetic differentiation in *N*. *melanorhinus*. As the loss of North American salt marsh habitat continues, anthropogenic barriers to dispersal may become more common and of larger effect. Changes that reduce dispersal among salt marsh sites are likely to decrease genetic diversity within populations and increase the risk of local extinction [51]. Habitat specialists, like those that rely on salt marsh habitat, are often the most susceptible to the effects of habitat loss. Species with lower levels of mobility are also at a higher risk than more mobile species like *N*. *melanorhinus*, which may be able to maintain dispersal across a more highly fragmented landscape. The significance of biogeographic changes on genetic variation should be of continued concern as our world is further shaped by anthropogenic habitat loss.

## Supporting information

S1 Table 2014 and 2015 Pairwise F_ST_ values based on AFLP markers as calculated in AFLPsurv. All pairwise F_ST_ values were significant (P < 0.05). Color gradient shows range of pairwise F_ST_ values in dataset, green the least divergent and red the most.
(TIF)

S1 Text Matrix of AFLP loci for 2014 individuals. (TXT)

S2 Text Matrix of AFLP loci for 2015 individuals. (TXT)

S3 Text List of 2014 sample’s hind femur lengths. (TXT)
