# Supplementary figures and images for: Population structure within the one-dimensional range of a coastal plain katydid
Source: PLoS One. 2017 Jun 9;12(6):e0179361. doi: 10.1371/journal.pone.0179361 (PMC5466309; doi:10.1371/journal.pone.0179361)

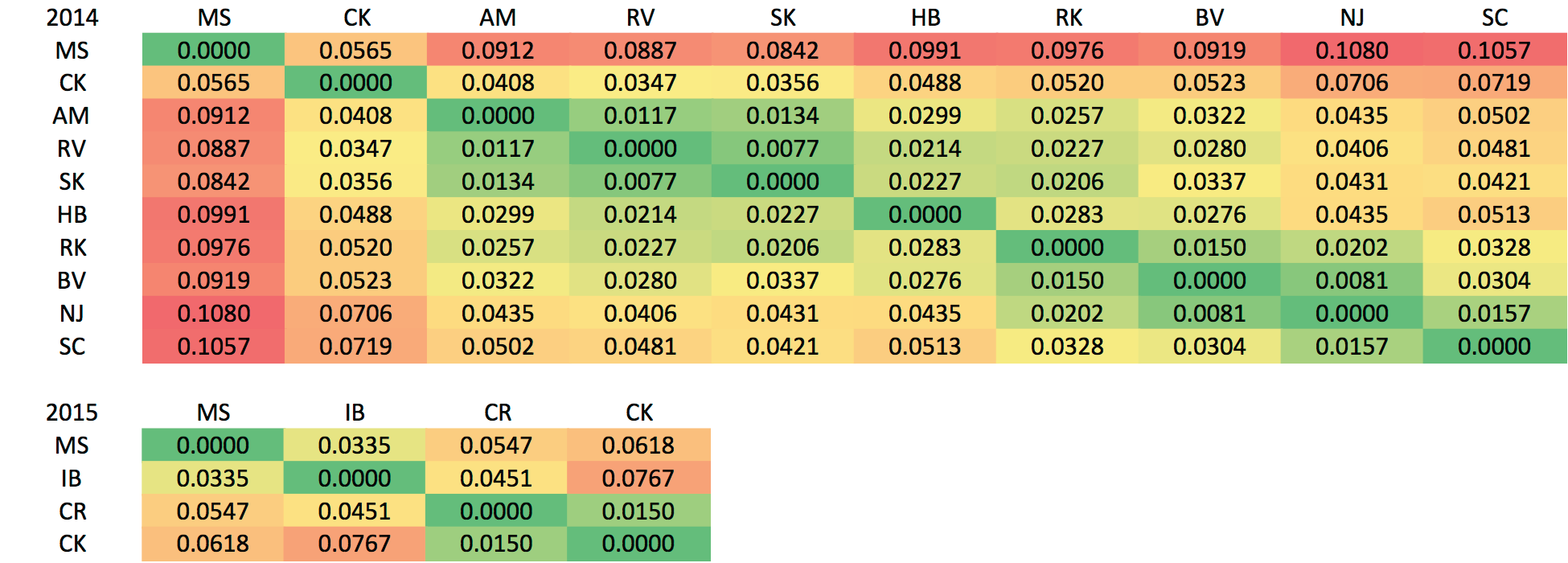

Supplement: S1 Table — All pairwise FST values were significant (P < 0.05). Color gradient shows range of pairwise FST values in dataset, green the least divergent and red the most. (TIF) [file pone.0179361.s001.tif]
